# Partial Phase‐Separation in the Initial Growth Stage of Exsolution‐Active SrTi_0.95‐x_Ni_0.05_Nb_x_O_3‐δ_ Thin Films

**DOI:** 10.1002/advs.76812

**Published:** 2026-08-03

**Authors:** Yen‐Po Liu, Moritz L. Weber, Dylan Jennings, Leonid L. Rusevich, Eugene Kotomin, Rainer Timm, Felix Gunkel, Regina Dittmann

**Affiliations:** ^1^ Peter Grünberg Institut (PGI‐7) Electronic Materials Forschungszentrum Jülich GmbH Jülich Germany; ^2^ Institute of Energy and Climate Research (IMD‐2) Materials Synthesis and Processing Forschungszentrum Jülich GmbH Jülich Germany; ^3^ Institute of Solid State Physics University of Latvia Riga Latvia; ^4^ Max Planck Institute for Solid State Research Stuttgart Germany; ^5^ Department of Physics Lund University Lund Sweden

**Keywords:** doped strontium titanate, epitaxial thin‐film, exsolution, initial‐stage nucleation, perovskite oxides, scanning tunneling microscopy, transmission electron microscopy

## Abstract

Heavily doped perovskite oxide thin films have emerged as versatile catalysts based on exsolution processes, which yield functional nanoparticles, but also introduce dopant inhomogeneities and internal phase separation. Here, we resolve the initial‐stage of thin film growth in heavily Ni‐doped and (Nb,Ni)‐co‐doped SrTiO_3‐δ_ utilizing scanning tunneling microscopy and spectroscopy (STM/S). While the growth of co‐doped SrTiO_3‐δ_ follows statistically distributed island nucleation, Ni‐doped SrTiO_3‐δ_ (STNi) exhibits a distinct pentagonal or hexagonal pattern of monolayer islands. A similar pattern is observed in thicker films by transmission electron microscopy (TEM). STS reveals Ni‐dopant clusters forming from the onset of the thin film growth and serving as nucleation points for embedded nanostructures at increasing film thickness. Quantitative analysis reveals that in the as‐grown monolayers, approximately 20% of the Ni ions segregate into nanoclusters, while the remaining ∼80% are dissolved in the perovskite lattice. Upon reduction (600°C, UHV), the entire Ni content of the sub‐monolayer STNi is found to nucleate as metallic nanoparticles at the surface, revealing two exsolution pathways from the perovskite lattice as well as from pre‐defined clusters. These findings provide deeper insights into the nucleation processes in heavily doped oxide and further reveal the role of dopant inhomogeneities for metal exsolution reactions.

## Graphical abstract

Phase separation emerges during the initial growth of heavily Ni‐ and Ni,Nb‐co‐doped STO thin films, leading to preferential accumulation of Ni and embedded NiOx nanoinclusions in thicker films. Nb co‐doping partially suppresses the ordering of these embedded structures, comprising about 20% of all Ni ions. Upon reduction, both phase‐separated and dissolved Ni contribute to the exsolution.

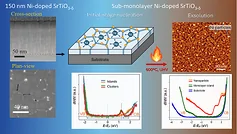

## 1 Introduction

Perovskite oxides, with the ABO_3_ structure, have been extensively studied and broadly applied across various fields, particularly in energy [1, 2, 3] and information technologies [4, 5, 6, 7, 8]. These epitaxial oxides, often studied in thin film form, can be tailored with thin film crystallinity and orientation, surface morphology, and electrostatic properties [9, 10, 11, 12, 13]. Within this class of materials, Ni‐doped SrTiO_3_ (STO), widely regarded as a typical model system for exsolution phenomena [14, 15, 16, 17], has garnered growing interest in recent years. To synthesize catalysts with a large nanoparticle density, large dopant concentrations (> 5at.%) are typically employed in the host oxides. Moreover, co‐doping of exsolution‐active cations and donor‐type dopants is often employed to enhance the electrical conductivity of the support oxide. However, heavy doping may lead to inhomogeneous cation distribution in ceramic oxides [18, 19, 20, 21, 22] and thin films [11, 14, 17, 23, 24, 25]. Interestingly, this inhomogeneous distribution can give rise to a partial phase separation, which has been observed in intentionally fabricated vertically aligned nanostructures (VANs) [26, 27, 28, 29, 30, 31, 32], as well as in exsolution‐active oxide perovskite thin films, which is known to be strongly entangled with the exsolution behavior [33]. The underlying processes leading to cation inhomogeneities and local phase separation, though commonly seen across the exsolution literature, remain insufficiently discussed due to characterization challenges.

In this work, we investigate the initial nucleation of heavily doped STO thin films and assess indications of phase separation and subsequent exsolution phenomena. Two key processes, (1) Ni segregation driven by growth kinetics and nucleation behavior in the initial growth phase, and (2) exsolution of metallic Ni driven by the subsequent reduction process. We employ controlled sub‐monolayer depositions and in situ scanning tunneling microscopy and spectroscopy (STM/S) analysis to resolve both the morphological features and their electronic states in the early‐stage growth for two differently doped strontium titanate systems, Ni‐doped STO (SrTi_0.95_Ni_0.05_O_3‐δ_, STNi) and (Ni, Nb)‐co‐doped STO (SrTi_0.9_Ni_0.05_Nb_0.05_O_3‐δ_, STNNi). While we find a stochastic nucleation of islands in the case of co‐doped STO, Ni‐doped STO reveals the emergence of a distinct surface pattern in its initial nucleation process, which correlates with lateral chemical inhomogeneities. The occasional appearance of dopant clusters suggests the early formation of a phase separation pattern. Consistently, co‐doped STNNi thin films appear to have denser embedded nanostructures, indicating a more homogeneous dopant distribution than in the thicker STNi, as revealed by scanning transmission electron microscopy (STEM). These contrasts, associated with chemical and structural variations, extend vertically through the oxide bulk and underscore the importance of understanding phase separation from the earliest stages of film growth. In Ni‐doped STO, STS (probing the local density of states (LDOS)) further indicates the occurrence of in‐gap states in the vicinity of the phase‐separated clusters, hinting toward the formation of locally confined defect clusters in this initial growth stage. Upon reduction, we finally demonstrate entire Ni exsolution from the sub‐monolayer STNi, confirming parallel exsolution pathways from both the pristine perovskite lattice (∼80%) and initially phase‐separated Ni‐ions (∼20%).

## 2 Results and Discussion

### 2.1 Doping‐Dependent Phase Separation of As‐Grown Thin Films

STNi and STNNi thin films were grown by pulsed laser deposition (PLD) in layer‐by‐layer growth mode. A representative characterization of a 50 nm thick film by AFM and XRD is shown in Figure S1c, demonstrating defined structural and morphological properties. Due to the doping, the thin films typically show an expanded lattice constant as compared to the STO substrate, which becomes evident as an intensity shoulder toward lower diffraction angles in 2θ‐ω scans (cf. Figure S1c). In contrast, based on the small sample volume and size of the NiO_x_ inclusions, however, direct detection via XRD remains challenging using lab‐based X‐ray sources. In the images, the surfaces are atomically smooth, while the XRD data reflect a high thin film quality with no secondary phases becoming evident in the investigated range, implying that a significant fraction of Ni‐dopants is incorporated into the perovskite oxide lattice according to the typical thin film characterization protocol. Corresponding STNi and STNNi thin films of 150 nm thickness are then studied by STEM using high‐angle annular dark‐field imaging (HAADF). In Figure 1, the STEM HAADF images of the STNi and STNNi films are presented in both the plan‐view (i.e., the viewing direction in the STEM is parallel to the film growth direction in (a), (c), (d), (f)) and the cross‐sections (i.e., the viewing direction is perpendicular to the film growth direction in (b), (e)). The plan‐view of STNi, Figure 1a, and STNNi, Figure 1d, show locally confined bright contrast, which can be identified as regions of high Ni content (cf. EDS analyses in Figure S2). The pattern of Ni‐rich regions in the STNi film consists of quite regular pentagonal or hexagonal features with sizes around 30 nm, forming a network. At triple junctions of this network, second‐phase NiO_x_ nanocolumns form (Figure 1c), which can be characterized as coherently embedded nanostructures that align along the vertical dimension similar to VAN structures, and Ni‐rich columns are observed to penetrate the full thickness of the film (Figure 1b).

**FIGURE 1:**
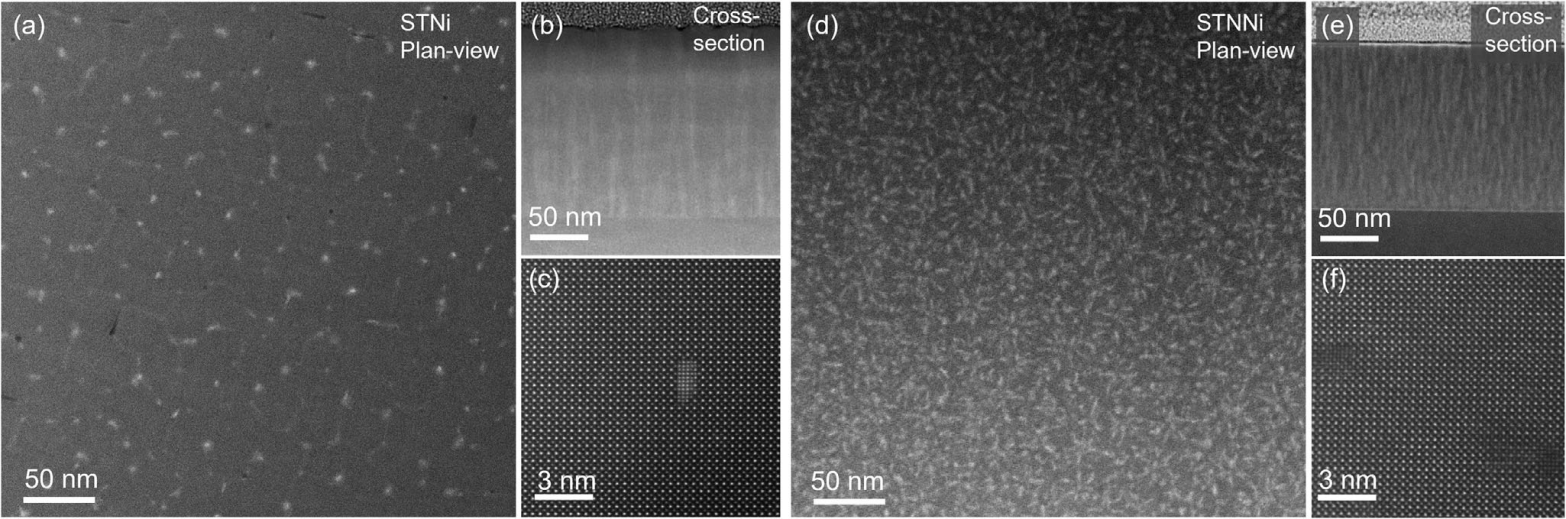
Comparison of as‐grown defect structures in the two 150 nm thick films by STEM HAADF images of STNi (a–c) and STNNi (e,f). (a) Plan‐view of the STNi sample. (b) Cross‐sectional image of the STNi film showing long‐range ordered embedded nanostructures. (c) High‐resolution plan‐view of the STNi film. (d) Plan‐view of the STNNi sample. (e) Cross‐sectional image of the STNNi film. (f) High‐resolution plan‐view of the STNNi film.

In comparison, plan‐view imaging of the co‐doped STNNi film (Figure 1d) shows a denser network of Ni‐rich features, and cross‐section imaging (Figure 1e) is consistent with a higher density, yet less regular pattern of Ni‐rich columns extending through the film. High‐resolution STEM imaging of the STNNi film (Figure 1f) shows that coherently embedded NiO_x_ secondary phases are present, consistent with previous reports [14]. Generally, columnar nanostructures start both from the bottom interface of the thin films as well as from intermediate depths, indicating that nucleation takes place already in the initial growth phase, but also continues during the growth. Further, the cross‐section images show tens to hundreds of nanometers vertical alignment of the inclusions for STNi, yet less extended and less ordered embedded nanostructures for STNNi, as shown in Figure 1b,e, respectively. Yet, the lateral distribution of these inclusions seems to significantly differ depending on doping.

### 2.2 STM Topography and Watershed Analysis

In order to study the thin film nucleation processes in the initial growth phase, samples of STNi and STNNi were fabricated with sub‐monolayer surface coverage. Here, an amount equivalent to less than one monolayer of STNi and STNNi is deposited employing RHEED‐monitored PLD, respectively. The benefit of this approach is that the morphology and assembly of perovskite islands can be studied before a coherent thin film layer is formed. The growth process was halted at the first minimum of the RHEED intensity oscillation, as shown in Figure S1d, observable due to the layer‐by‐layer growth mode [12, 33, 34]. The sub‐monolayer STNi was deposited onto the Nb‐doped STO single‐crystal substrates, which provide the electronic conductivity required for subsequent STM analysis. This procedure is expected to deposit roughly 50% coverage of the substrate surface, while the absolute coverage expected at the RHEED intensity minimum may depart from 50% of coverage as it is determined by the maximum step‐density [35] during monolayer growth. Such a behavior has been observed, e.g., for non‐stoichiometric STO thin films [36], making it likely that different doping may influence the nucleation behavior and thus the resulting coverage around the initial RHEED minimum. CAFM was used first to determine the surface quality and to test the surface conductivity by performing IV spectra to determine ideal STM tunneling parameters (see Figure S4). Although Nb‐doped STO (100) is widely used as a conductive substrate due to its excellent electrical conductivity, STM/STS investigations are not trivial because surface effects such as surface termination and surface band‐bending can lead to a depleted near‐surface region [37, 38].

The substrate morphology prior to the deposition is atomically smooth, as shown in Figure S4. After sub‐monolayer deposition of STNi, a large STM image (300 × 300 nm^2^), presented in Figure 2a, reveals three different atomic terraces. Corresponding scan profiles along the red and blue lines are shown in Figure 2b,c, respectively. The red line profile exhibits an 8 Å step height, indicating a 2‐unit‐cell step height resulting from step bunching, commonly observed in Nb‐doped STO substrates that exhibit an increased miscut angle [39]. We note that the phase separation takes place on top of each atomic step terrace. Therefore, terrace width, step‐size, or potential step bunching observed on a larger scale are not expected to influence the observed behavior. The blue line profile, a line scan on a single terrace, shows a step‐like variation of 4–5 Å, corresponding to about one unit cell of STO. The elevated regions are hence identified as film islands, while the lower regions correspond to uncovered substrate, denoted as trenches. The thin film islands thus partially cover the substrate as expected for a sub‐monolayer deposition. This is clearly visible in Figure 2a inset, where nanofeatures appear on each atomic step. For comparison, the STNNi film shown in Figure 2d also exhibits three different atomic terraces. The scan profiles along the orange and green lines are displayed in Figure 2e,f, respectively. The orange profile in (e) shows two 4 Å‐high steps indicating single‐unit‐cell step heights. The green profile in (f) reveals trenches of approximately 4 Å in depth, comparable to the features in the blue profile in (c).

**FIGURE 2:**
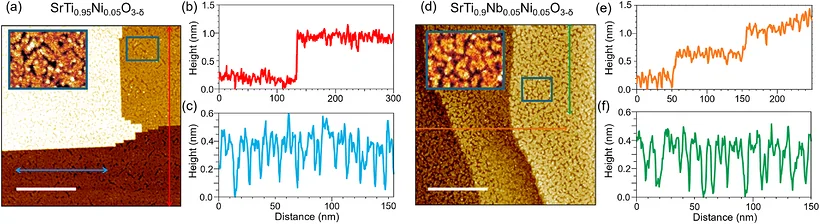
STM topography of (a) STNi and (d) STNNi and their scan profiles, with both scan sizes of 300 nm by 300 nm. The white scale bars are 100 nm. Close‐up images extracted from the dark blue rectangle are shown in the insets. (b,e) Scan profiles, averaged over 3 nm width, across the step edges of the substrates. (b) Two‐unit‐cell steps show a height difference of around 8 Å, (e) two individual single‐unit‐cell steps show about 4 Å step height each. (c,f) Scan profiles, averaged over 0.5 nm width, on the step edges, showing the film thickness, or called trench depth, from the surface to the substrate, about 4 Å. The scanning parameters of the images (a) and (d) are *V_T_* = +2.35 V, *I_T_* = 20 pA and 6.5 pA, respectively.

Both samples display a surface that is partially covered by film islands of 1 unit cell in height, while the apparent film coverages are around 75%, which is higher than the targeted 50% coverage. The reasons could be either a result of the convolution of the tip and the sample, or because the film is actually deposited with a higher amount than 0.5 unit cell, which may be related to the offset between RHEED intensity minimum and 50% surface coverage, as reported in the literature [12, 34, 36, 40]. Notably, the film morphologies in Figure 2a,d show distinct differences, particularly in the trench structure surrounding the monolayer islands after initial nucleation. STNi tends to form elongated, linear trenches approximately 20 nm in length; in comparison, STNNi exhibits shorter and less ordered features. A higher‐resolution STM analysis on a single atomic terrace is used to further resolve these nanostructures and clarify the differences in surface morphology.

Corresponding topography images zoomed in on flat terraces of both STNi and STNNi films are shown in Figure 3a,d, respectively. In Figure 3a, monolayer islands with dark trenches (substrate) plus additional clusters with larger height become apparent, indicated with light blue arrows. To systematically study the characteristic morphology, a watershed algorithm with a height threshold of 4.1 Å is applied to Figure 3a, color‐coding connected monolayer islands, as shown in Figure 3b. The regular, differently colored shapes in Figure 3b correspond to individual monolayer islands. The colors are assigned arbitrarily and serve only to visually separate individual islands.

**FIGURE 3:**
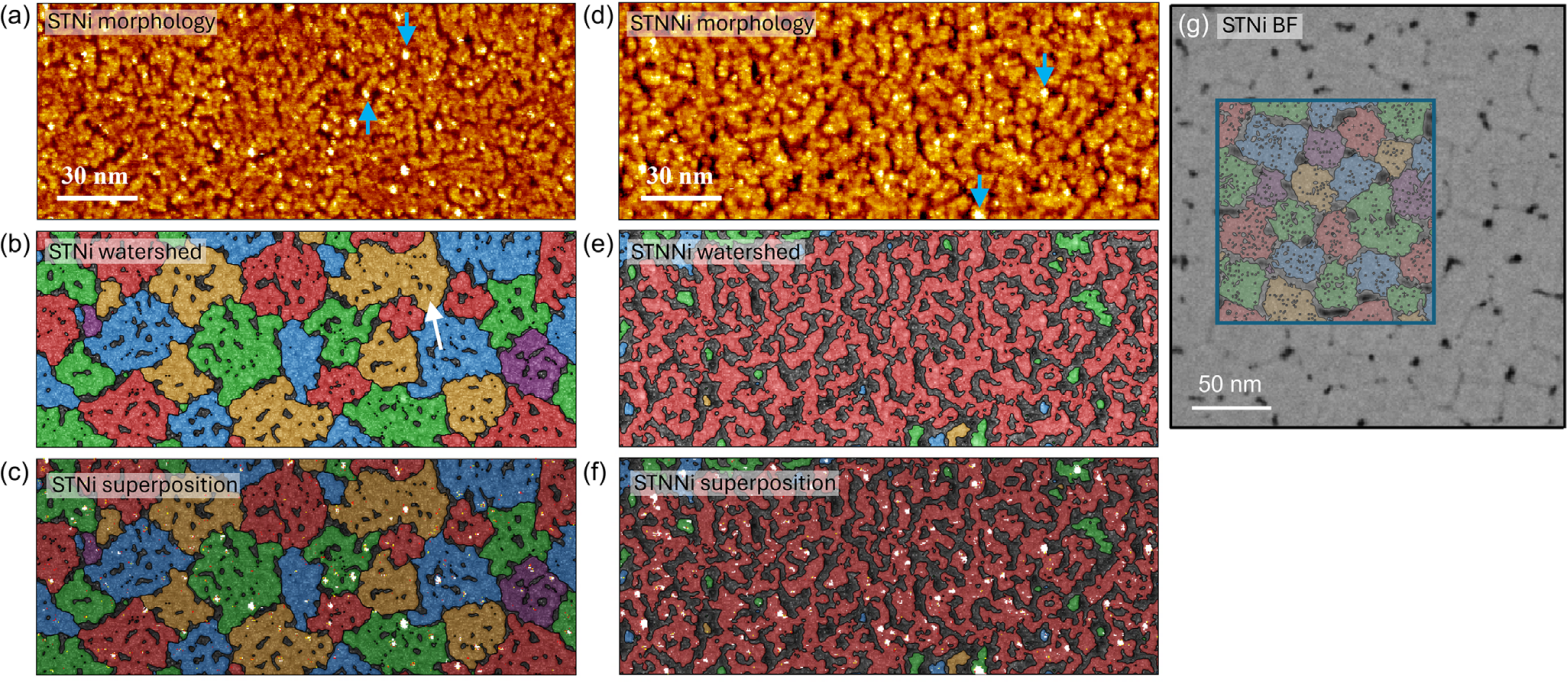
STM topography of the two samples. (a–c) STNi (d–f) STNNi. (a,d) STM images of 200 nm by 80 nm show different preferred trench behaviors and clusters apparent by bright contrast, indicated with light blue arrows. (b,e) Watershed analysis merging the islands that have physical connections, separated by the trenches, using a height threshold of 4.1 Å. The white arrow in (b) marks a larger island possibly joined by a cluster residing between neighboring islands. (c) Overlap of the topography (a) with emphasis on the clusters and watershed analysis (b) to show the location of clusters. (f) Overlap of the topography (d) with emphasis on the clusters and watershed analysis (e) to understand the location of clusters. (g) Identical watershed analysis of the bright‐field STEM image of 150 nm thick STNi.

Interestingly, the surface morphology reveals additional decorations, forming clusters that exceed the expected height of a single unit cell. To analyze the appearance of those clusters in detail, a superimposed image of Figure 3a,b is presented in Figure 3c. In the image, the brighter spots indicate clusters, which exhibit full‐width half‐maximum (FWHM) diameters around 2.5 to 3.5 nm, analyzed in detail in Figure S5a,b. Similarly, the embedded cluster diameter determined for thicker films by STEM is also about 1.5–3 nm, as shown in Figure S5c,d. The slightly larger diameter defined by STM is likely due to the tip convolution. In STNi, all such clusters appear next to the trenches, predominantly at the edges of the monolayer islands formed in this initial growth phase. This is intrinsically connected to the mechanisms governing initial thin film nucleation.

In comparison, the topography of the STNNi film, shown in Figure 3d, also shows trenches, monolayer islands, and clusters, but the cluster size is much smaller, and the trenches are not as defined as in the STNi case. The same watershed algorithm with the same height threshold as for the STNi analysis is applied and shown in Figure 3e, and no regular island correlating to the trench locations appears here, implying that the film nucleation is more interconnected and homogeneous in its initial stage. An image with a color scale emphasizing the topmost surface morphology (white, as clusters) of the STNNi image is plotted in Figure 3f, where no clear regular features appear. This is also consistent with the structure of the 150 nm thick STNNi film imaged by STEM, as shown in Figure 1d and Figure S2b.

The regular pattern revealed by STM in the initial growth stage is hence consistent with the subsequent formation of extended embedded nanostructures in thicker films. Figure 3g shows the same watershed analysis applied to the bright‐field STEM plan‐view for STNi. Interestingly, each regular shape from STM topography has a size and shape that closely matches the pattern of Ni distribution observed in the 150 nm thick film in Figure 1a and Figure S2a. The mean area of these islands determined by Mountains 9 watershed analysis is 494 ± 35 nm^2^ for STM on sub‐monolayer islands and 526 ± 39 nm^2^ for TEM on the thin film. By the assumption that these features are regular pentagons, these areas correspond to an average diagonal of 27.4 ± 1.1 and 28.3 ± 1.1 nm, respectively.

### 2.3 Local Electronic Structure Analysis

STM has already been used to image surface reconstructions in (110) Nb:STO [41, 42, 43] as well as (100) Nb:STO using in situ cleaving techniques [44, 45], showing how facile electronic structure responds to slight changes in surface chemistry. At the same time, STS analysis is scarcely used, presumably due to the significant bias voltages required. The above STM results reveal distinct nucleation behaviors of monolayer islands and cluster formation between STNi and STNNi, indicating different initial phase separation mechanisms. Here, we investigate the electronic structure of the nanostructures by STS at peculiar positions, which may be related to the local stoichiometry.

A zoom‐in 100 × 100 nm^2^ STM image is performed at a neighboring area for investigating STS. Scanning tunneling spectra are obtained at specific locations indicated with arrows in Figure 4a, and the resulting *dI/dV‐V* spectra are shown in Figure 4b, while the original *I*(*V*) curves are included as insets. The *dI/dV‐V* spectra of the monolayer islands are plotted as blue and orange curves. Linear fits are applied to the valence band (VB) and conduction band (CB) near the bandgap region, and the *x*‐axis intercepts of these fits are taken as the band edges, in agreement with the literature [46, 47]. The extracted valence band maximum and conduction band minimum are positioned 1.8 and 1.5 eV relative to the Fermi level, resulting in a total bandgap of 3.3 eV, consistent with the reported ∼3.2 eV bandgap of pure STO [48, 49, 50]. It is worth mentioning that the surface states on the film may have contributed to the Fermi‐level pinning effect (or space charge effects) [37, 38], resulting in the Fermi level located closer to the center of the bandgap than in the bulk, and that *E_F_* corresponds to the zero‐bias condition in all STS spectra. For the phase‐separated clusters at the edges of the monolayer islands, the valence band edge overlaps well with the film spectra, while the conduction band edge shifts by 0.4 eV to a position 1.9 eV above the Fermi level, leading to a total bandgap of 3.7 eV. More interestingly, a well‐defined in‐gap state at∼1.2 eV above the Fermi level is observed. The in‐gap state may indicate a complex defect cluster in the perovskite.

**FIGURE 4:**
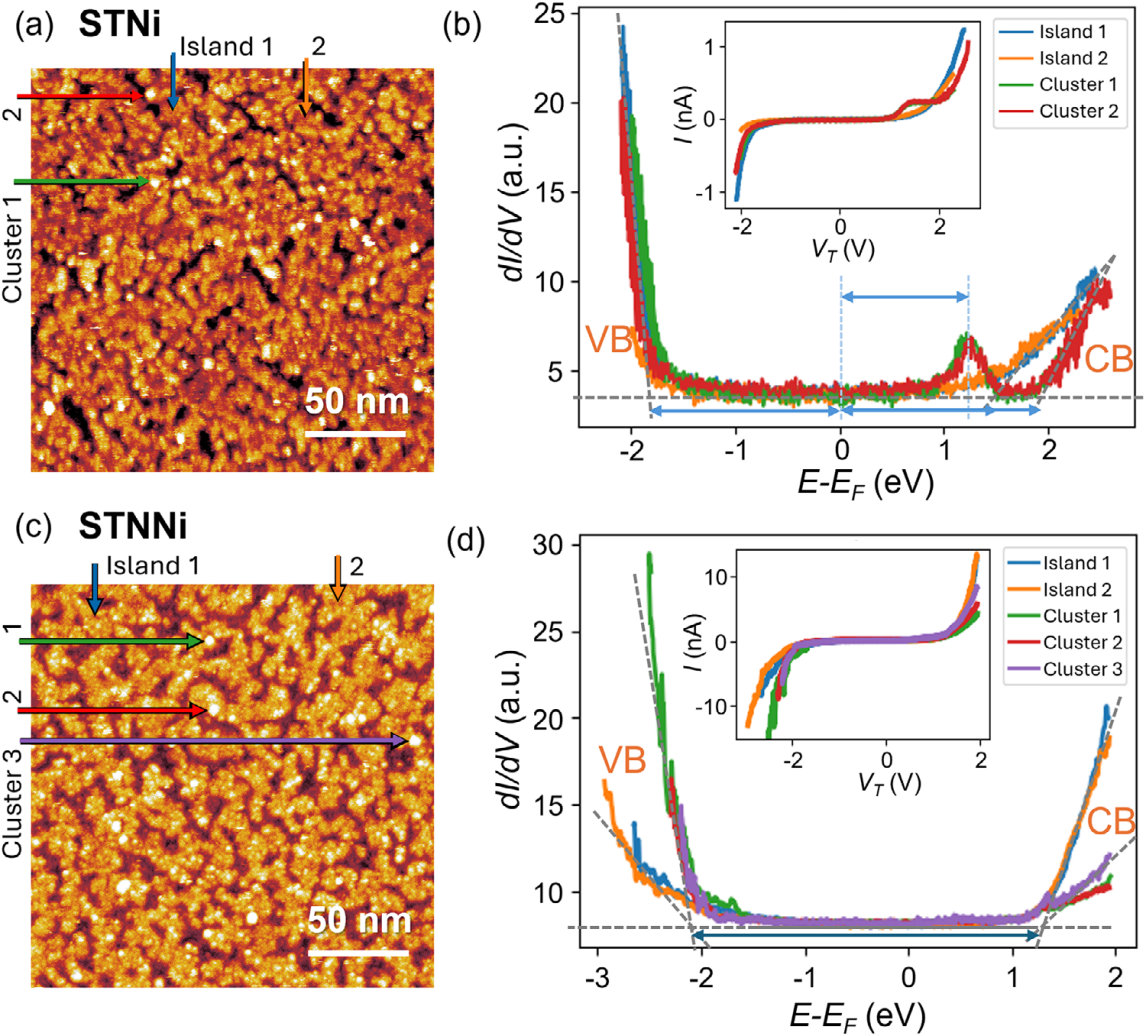
Spatially resolved STS. (a) STM image of the sub‐monolayer STNi with indicators showing the locations where the *dI/dV‐V* spectra shown in (b) are obtained. (c) STM image of the sub‐monolayer STNNi with indicators showing the locations where the *dI/dV‐V* spectra shown in (d) are performed. The white scale bars are 50 nm.

In STM/S, a positive sample bias corresponds to electrons tunneling from the tip into empty states of the sample. Accordingly, depleted acceptor states are seen in STS data between the Fermi level and the CB onset [51], as illustrated in Figure S6. The in‐gap state observed in spectra taken at a cluster position can therefore be assigned to an acceptor level, consistent with the expected role of Ni 3d states in the lattice. Comparable spectral features have been reported previously in systems with dominant Ni‐rich doping [52, 53]. To qualitatively assess the distinct in‐gap state in the case of STNi, we consider the Ni dopants, which are, in the simplest case, expected to substitute for Ti on the perovskite B‐site, forming doubly charged 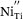 acceptor states [54]. To maintain charge neutrality, these acceptor states are typically compensated by the formation of oxygen vacancies [55, 56, 57, 58, 59]. Density functional theory (DFT) calculations for several configurations in which Ni partially replaces Ti are discussed in detail in Figure S7. Notably, clear in‐gap states appear above the valence band maximum when Ni^2+^ replaces 12.5% Ti^4+^on the B‐site, leaving 4.17% oxygen vacancies, $V_{O}^{\cdot \cdot }$, for both first nearest neighbors (1NN) and spatially separated 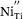 and $V_{O}^{\cdot \cdot }$, within the unit cell, as shown in Figure S7a,b, respectively. Further calculations presented in Figure S7c considering Ni^4+^ replacing Ti^4+^ without oxygen vacancies [60] show in‐gap states closer to the conduction band minimum as donors, hence a less likely case compared to the 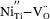 cases. The calculations hint that the presence of a 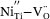 complex defect cluster introduces extra energy states (bands) within the band gap, narrating the link between the observed in‐gap states and the observed defect clusters. We note that DFT cannot give an unambiguous identification of the origin of the observed in‐gap states, but may yield a qualitative assessment on potential defect structures in the vicinity of the NiO_x_ inclusion can yield the observed in‐gap states. 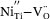 may be more likely to occur in Ni‐only doped parent compound, which requires charge compensation of Ni dopants via oxygen vacancies.

On the other hand, the larger bandgap than expected for strontium titanate may match the band gap of NiO, which is reported to be 3.65–3.88 eV [52, 53, 61, 62, 63, 64, 65]. Fast Fourier transformation (FFT) analysis of high‐resolution plan‐view images (Figure S8) suggests that rocksalt NiO_x_ is present as inclusions in both STNi and STNNi films. Combining this information, the spectral features of phase‐separated clusters may likely represent a convolution of contributions from the NiO_x_ and 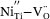 complex defect clusters. The presence of an additional electronic state in a wider bandgap, in combination with the observed nanostructure of the film, strongly suggests that these phase‐separated clusters appear to serve as nuclei for the embedded nanoclusters observed in thicker films. This interpretation is further supported by energy‐dispersive X‐ray spectroscopy (EDS) results, which reveal strong Ni and O signals for the embedded phase [66], as shown in Figure S2a. However, accurately modeling the atomic structure of these features remains challenging, as DFT simulations rely on periodic unit cells and differ marginally for monolayers with defects.

In the spectra of the STNNi shown in Figure 4d, performed at the locations indicated in the STM image of Figure 4c, the monolayer shows a comparable bandgap of about 3.4 eV, where the VB maximum is 2.1 eV below the Fermi level while the CB minimum is 1.3 eV above the Fermi level. The clusters observed for STNNi interestingly have a very similar fitted bandgap as the surrounding monolayer. The bandgap is consistent with pure STO and comparable to the one observed for the STNi islands (Figure 4a). Here, the *dI*/*dV*‐*V* spectrum does not show distinct peaks within the bandgap. Still, from the difference in the spectra at different positions, it is clear that the clusters have denser states, i.e., a steeper slope, close to the VB onset, while the monolayer has a lower density of states. The situation is opposite in the CB, where the clusters have a lower density of states close to the band onset than observed on the monolayer. Moreover, a shoulder is evident at the VB maximum, and the entire spectrum is shifted slightly toward *n*‐type (the CB edge lies closer to *E_F_* than the VB edge), as compared to the spectra in Figure 4b. Such characteristics resemble the typical signature of *n*‐type doping [51], likely introduced by the donor Nb doping. This implies that the clusters and the film are slightly different in composition. Nevertheless, the cation and anion distribution across the film appears more homogeneous than that of STNi, based on the evaluation of the local bandgap. The absence of in‐gap signatures may further be related to the co‐compensation of Nb‐ and Ni‐dopants, resulting in a lower concentration of associated oxygen vacancies, required to form the suspected 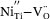 defect clusters discussed in STNi.

Given the combination of STM and STEM analysis of the thin films and the DFT calculations, a phase separation mechanism hypothesis can be made. During the initial sub‐monolayer growth, Ni cations preferentially segregate to the edges of STNi islands. During further deposition, the surface coverage increases, which results in the monolayer islands merging into a coherent film. At the triple junctions between the islands, enough Ni is concentrated that 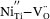 defect clusters form and a NiO_x_ second‐phase nucleates. The interconnected networks of Ni‐rich strontium titanate (see Figure 1a) are considered to be residues from the segregation of Ni to the edges of the islands, leaving behind a “fingerprint” of the thin film growth islands.

In addition to the planar distribution, the vertical distribution in STNNi also exhibits denser but shorter and less ordered features both laterally and vertically (Figure 1d,e and Figure S5) compared with STNi. The origin of these internal structural differences between the STNi and STNNi thin films are as‐of‐yet unclear. One possible explanation may be that the addition of Nb to the system stabilizes Ni in the perovskite lattice, limiting the segregation of Ni cations to the island edges. In support of this hypothesis, it has been suggested in the literature that compensating acceptor and donor co‐doping can increase dopant solubility in complex oxides [67, 68]. From the perspective of ionic radius for B‐site elements, Ni (II) has a larger ionic radius compared to Nb (V), followed by Ti (IV) [23, 69]. Therefore, this ionic‐size co‐compensation effect of incorporating both smaller and larger dopants into the perovskite structure may help to stabilize the film structure and suppress the formation of nanocolumns, resulting in a more homogeneous morphology in the STNNi films.

### 2.4 Exsolution From the Sub‐Monolayer STNi

We proceed to examine the exsolution behavior from the STNi sub‐monolayer under thermal treatment, using STNi, showing the more pronounced signature of initial phase separation. The sub‐monolayer STNi is reduced by UHV annealing with a base pressure of 10^−11^ mbar, as detailed in the Experimental Details. No significant changes in surface morphology or electronic structure were observed in either STM or STS following annealing at 400°C for 20 min, as shown in Figure S9. However, annealing at 600°C for 10 min induces a clear morphological change.

Following annealing at 600°C in UHV, the surface morphology exhibits a clear transformation, as shown in Figure 5a. A larger‐scale image illustrating features across multiple atomic steps is provided in Figure S10. The 4.1 Å threshold watershed analysis, shown in Figure 5b, further confirms that the trench structure, initially formed the pentagonal and hexagonal patterns, becomes less pronounced, indicating island coalescence during the thermal treatment. Concurrently, brighter spots corresponding to exsolved particles emerge with enhanced contrast and increased density, with diameters ranging from 3.5 to 6.5 nm. A particle coverage of 1.1% of the surface area is determined using the watershed analysis with a height threshold of 5.5 Å (features higher than the surrounding film). Note, however, that the measured particle size and coverage are influenced by tip convolution with the actual particle shape, resulting in a broadening of the apparent topographic features. The observed particle size is in agreement with previous STEM and AFM studies [66, 70, 71] on exsolution particles in the same material system, in the applied annealing time and temperature range.

**FIGURE 5:**
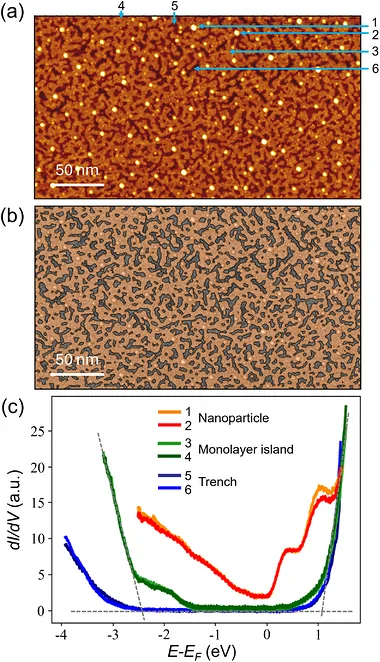
The sub‐monolayer STNi after exsolution by annealing in UHV at 600°C. (a) STM image. (b) Identical watershed analysis applied to the area. (c) *dI/dV‐V* spectra of nanoparticles, monolayer islands, and trench.

The *dI/dV* spectra of the exsolved particles (positions 1,2), film (positions 3,4), and trench (positions 5,6) are performed after annealing, as shown in Figure 5c. The red and orange curves in Figure 5c, taken from relatively large particles, exhibit metallic behavior, with no non‐conducting area at a lower bias. The Fermi level shifts toward the conduction‐band minimum at all measured locations upon annealing, consistent with improved band alignment with the Nb‐doped STO substrate [38]. Notably, the spectra acquired from the exsolved particles show distinct oscillations at approximately +0.35 and +1.1 V within the conduction band. This phenomenon can be regarded as a quantum well phenomenon resulting from the confinement of the metallic nanoparticles. By assigning the first quantized energy level *E_n = 1_* is at +0.35 eV and using the estimation equations provided in the Supporting Information, the corresponding quantum dot diameter is calculated to be approximately 5 nm. This value is in good agreement with both the Ni particle size observed in the topography and the particle size selected to perform STS.

The STS on the monolayer islands after exsolution shows notable differences from the spectra acquired prior to Ni exsolution, consistent with observations reported in other exsolved systems [72]. After Ni exsolution out of the lattice, the spectra of the sub‐monolayer film (green, Figure 5c) reveal a bandgap of 3.5 eV, along with a distinct shoulder near the valence band maximum at ‐2 eV, which is a typical *n*‐doping behavior in STS of semiconductor materials [51]. It is worth noting that reduced, unintentionally doped SrTiO_3‐δ_ commonly exhibits weak *n*‐type conductivity [59, 73]. Moreover, the same reduction process that leads to Ni exsolution also leads to high $V_{O}^{\cdot \cdot }$ concentration in STO, which then appears as an n‐type state in the spectra [74, 75]. As a comparison, the STS of the trench is shown as the blue curves in Figure 5c, which indicates an even larger bandgap.

Quantitative estimation of the exsolved Ni amount is performed under the assumption that the particles exhibit a semi‐ellipsoidal geometry, serving as an approximation for the real particle shape, with a volume that can be expressed as $V=\frac{2}{3}\;\pi r^{2}h$. Detailed quantitative calculations for theoretical number and experimental cases are presented in the Supporting Information. The total number of Ni atoms within a representative 200 × 200 nm^2^ area is derived by multiplying the total particle volume by the atom density of metallic Ni. Here, the cumulative projected area of all particles within this region (*n*  ×  π*r* ^2^) corresponds to the experimentally determined surface coverage of 1.1% × 200 × 200 nm^2^, and the average particle height *h* is extracted from the STM scan profile, approximately 4 Å. Using the bulk Ni density, the total number of exsolved Ni atoms in this area is estimated to be approximately 10k atoms. This value closely matches the theoretical number of Ni atoms, 10k, expected for a 5% Ni‐doped STNi with 75% sub‐monolayer coverage, suggesting that nearly all initially deposited Ni ions accumulate in the form of nanoparticles after the reduction. The result indicates a full Ni diffusion to the surface achieved upon exsolution, which is consistent with the expectation that the kinetics of bulk mass transport does not pose a considerable constraint for nanoparticle exsolution in the present monolayer‐thick STNi sample.

Based on the estimated amounts of Ni present in clusters detected at the surface of as‐deposited films as compared to the amount of Ni detected in metal nanoparticles after thermal reduction, we estimate the relative proportions of Ni incorporated in the lattice and Ni aggregated as the second‐phase. From the same quantitative evaluation as applied for Ni exsolution based on the STM results (see detailed calculations in the Supporting Information), an upper limit of roughly 2k Ni atoms is initially present as distinct nanoclusters within the 200 × 200 nm^2^ area, assuming that all clusters correspond to fully phase‐separated NiO_x_. Given a total of ∼10k Ni atoms in this area, estimated by both theoretical and experimental evaluation, the ∼2k Ni atoms contained in nanoclusters correspond to approximately 20% of the nominal 10k Ni content, whereas the remaining ∼80% of Ni ions are thus incorporated into the monolayer perovskite lattice.

## 3 Conclusion

This study investigates the doping‐dependent partial phase separation in initial‐stage PLD‐grown STNi and STNNi thin films utilizing STM/S supported by STEM. Our findings reveal that the nucleation process at the initial stage of growth results in a distinct in‐plane pattern of Ni‐rich nanoclusters, which may later on yield embedded nanostructures in thicker films, dictated by their effective doping. In STNi, the network‐like regular pattern arises from Ni cations preferentially segregating to island step edges, collocated at the trenches between islands right after nucleation. In contrast, Nb co‐doping may partially suppress NiO_x_ segregation toward the island edges in the initial growth phase, leading to less pronounced NiO_x_ accumulation at island triple junctions and a less ordered formation of embedded nanostructures in the subsequent growth process.

Upon thermal annealing, Ni exsolves from sub‐monolayer STNi, emerging as homogeneously distributed metallic Ni particles on the surface, accompanied by the disappearance of the initial pentagonal and hexagonal patterns. Quantitative analysis indicates that, at the solubility limit of 5% Ni‐doping in STO, approximately 20% of the Ni formed complex defect clusters in the as‐grown film, while the remaining 80% resided within the perovskite lattice. Both forms of Ni contribute to the exsolution, demonstrating parallel exsolution pathways from pre‐nucleated clusters as well as from the pristine lattice.

Overall, these findings provide fundamental insights into the interplay between dopant segregation, phase separation, and initial‐stage nucleation in heavily doped STO systems. The study enhances control over phase separation phenomena in complex oxide thin films, offering valuable guidelines for designing functional oxide thin films with tailored properties for applications in catalysis, electronics, and energy devices.

## 4 Experimental Section

### 4.1 In Situ Transfer and Pre‐Characterization

The PLD chamber is connected to other ultrahigh vacuum (UHV) instruments for in situ characterizations. These include a Scienta Omicron VT SPM (offering in‐situ AFM and STM) and a vacuum suitcase, which can keep the vacuum in low 10^−9^ mbar for up to one week.

An in situ conductive atomic force microscopy (cAFM) experiment using a PPP‐CONTPt cantilever (Nanosensors, Neuchatel, Switzerland) in contact mode with a setpoint of 2 nN was performed using the Omicron SPM to check the surface morphology (as shown in Figure S4a,b) and the conductivity parameters (as shown in Figure S4c) for LT‐STM measurements. Following the preliminary characterization, the vacuum suitcase was used to transport the sub‐monolayer samples to the LT‐STM for high‐resolution measurements in a UHV environment, without breaking the vacuum.

### 4.2 Pulsed Laser Deposition (PLD)

The STNi and STNNi films are grown by PLD and monitored by reflection high‐energy electron diffraction (RHEED) on [001] orientated 0.5% Nb‐doped SrTiO_3_ substrates (Shinkosha Co. Ltd., Yokohama, Japan). The epitaxial PLD is performed with a Surface PLD workstation (SURFACE systems + technology GmbH & Co. KG) at a substrate temperature of 650°C (measured on the backside of the sample holder) using a 925 nm IR‐diode laser, a target ablation repetition rate of 5 Hz using a 248 nm KrF excimer laser, a laser fluence of 1.14 Jcm^−2^, a target‐to‐substrate distance 57 mm, and an oxygen pressure of 0.110 mbar. These growth conditions correspond to an average growth rate of approximately 6.5 s per monolayer for all deposited thin films. The samples were then quenched down to room‐temperature directly after deposition. For reference, 50 nm thick films are grown (layer‐by‐layer type signatures recorded in Figure S1a) and characterized using atomic force microscopy (AFM, Cypher, Oxford Instruments Asylum Research Inc., Santa Barbara, USA) and X‐ray diffraction (XRD, D8 Discover, Bruker AXS GmbH, Karlsruhe, Germany), as shown in Figure S1c,d, respectively. The same deposition procedure is then applied for 150 nm thick films and sub‐monolayer depositions.

### 4.3 Scanning Transmission Electron Microscopy (STEM)

STEM analysis of 150 nm thick thin films was performed to analyze the atomic‐scale structure and composition of the STNi and STNNi. STEM imaging and EDS mapping were done using a FEI Titan G2 80–200 ChemiSTEM. Supplemental EDS mapping was done using a Hitachi HF5000 S/TEM. Plan‐view and cross‐section TEM lamellas were prepared from each sample using an FEI Helios NanoLab 460F1. Plan‐view lamellas were prepared using a similar process as described in O'Shea et al. [76]. EDS data analysis was performed using HyperSpy [77].

### 4.4 Low Temperature Scanning Tunneling Microscopy and Spectroscopy

The CreaTec LT‐STM operates at 5 K under UHV, below 1 × 10^−10 ^mbar, and the STM piezo parameters are calibrated with an Au (111) crystal, using atomic step heights and atomic resolution within herringbone surface construction. STM tips are electrochemically etched from 0.38 mm diameter tungsten (W) wire in 2 M NaOH solution using an applied voltage of 3 V and a threshold current of 1.3 mA. STS is verified by confirming the presence of the characteristic ‐400 mV extra state on the Au (111) surface. The STM bias is set according to the IV spectra of cAFM measurement at room‐temperature, which shows the threshold voltages of SrTiO_3_, later defining the tunneling bias region for STM. To stabilize the STM scanning on such high bandgap materials, the tip bias is trained with a high bias on an Au crystal, i.e., +2.35 V. The tunneling current setpoint for approaching is 130 pA and for scanning is 30 pA. The same tip as for the STM imaging of STNi is conditioned on Au 111 crystal, as shown in Figure S11.

To conduct the STS measurement, STM is performed first with a higher tunneling current setpoint, and then, at specific interesting locations, the scanning is interrupted, and the tunnel bias is ramped while measuring the resulting current. By modulating the bias with an AC signal of 70–100 mV amplitude at 313 or 381 Hz and using a lock‐in amplifier, the differential conductance *dI/dV* is measured simultaneously, indicating the local density of states (LDOS) [51, 78]. An illustration of the mechanism for STS mapping LDOS in the case of positive or negative sample bias is shown in Figure S6.

### 4.5 Sample Annealing in UHV

Metal exsolution of Ni from the STNi sample was performed at an elevated temperature in the preparation chamber of the LT‐STM. The process is first performed at 400°C for 20 min, for which the results are shown in Figure S9. Subsequently, the sample was further annealed to 600°C for 10 min. The base pressure is 9 × 10^−11^ mbar at room‐temperature, and it was raised to 2 × 10^−9^ mbar at an annealing temperature of 400°C and 1.3 × 10^−8^ mbar at an annealing temperature of 600°C.

### 4.6 STM Topography Watershed Analysis

Watershed analysis uses a watershed algorithm to separate different continuous objects in the STM images, with a threshold height. The zero‐level is set at the lowest point of the whole image; therefore, a higher threshold number than the expected height of a single unit‐cell step (i.e., lattice constant) may be needed due to a random lowest point in a scan. This watershed analysis is conducted using the “grain analysis” function in threshold detection mode provided by Mountains 9. A diameter filter ranging from 1 to 50 nm is applied to exclude islands that resulted from noise or several islands merged.

## Author Contributions

**Leonid L. Rusevich**: formal analysis, Writing – review and editing. **Moritz L. Weber**: conceptualization, methodology, validation, data curation, writing – review and editing. **Rainer Timm**: writing – review and editing, formal analysis. **Regina Dittmann**: writing – review and editing, supervision, validation. **Felix Gunkel**: writing – review and editing, supervision, data curation, validation. **Eugene Kotomin**: writing – review and editing, formal analysis. **Yen‐Po Liu**: conceptualization, methodology, investigation, validation, visualization, data curation, formal analysis, writing – original draft. **Dylan Jennings**: investigation, methodology, visualization, writing – review and editing, formal analysis.

## Conflicts of Interest

The authors declare no conflicts of interest.

## Supporting information




**Supporting File**: advs76812‐sup‐0001‐SuppMat.pdf.

## Data Availability

The data that support the findings of this study are available from the corresponding author upon reasonable request.
