# Supplementary material for: Partial Phase‐Separation in the Initial Growth Stage of Exsolution‐Active SrTi0.95‐xNi0.05NbxO3‐δ Thin Films
Source: Adv Sci (Weinh). 2026 Aug 3:e76812. Online ahead of print. doi: 10.1002/advs.76812 (PMC13431241; doi:10.1002/advs.76812)
Supplement: Supplementary file 1 — Supporting File: advs76812‐sup‐0001‐SuppMat.pdf. [file ADVS-9999-e76812-s001.pdf]

Supporting information

Partial Phase-separation in the initial growth stage of exsolution-active $SrTi_{0.95-x}Ni_{0.05}Nb_xO_{3-\delta}$ thin films

Yen-Po Liu[1], Moritz L. Weber[1,#], Dylan Jennings[2,^], Leonid L. Rusevich[3], Eugene Kotomin[3,4], Rainer Timm[5], Felix Gunkel[1], Regina Dittmann[1]

[1] Peter Grünberg Institut (PGI-7), Electronic Materials, Forschungszentrum Jülich GmbH, 52428 Jülich, Germany

[2] Materials Synthesis and Processing (IMD-2), Institute of Energy and Climate Research, Forschungszentrum Jülich GmbH, 52428 Jülich, Germany

[3] Institute of Solid State Physics, University of Latvia, 1063 Riga, Latvia

[4] Max Planck Institute for Solid State Research, 70569 Stuttgart, Germany

[5] Division of synchrotron radiation research, Lund University, 221 00 Lund, Sweden

#now at: Kyushu University, Next-Generation Fuel Cell Research Center, 744 Motooka, Nishi-ku, Fukuoka, 819-0395, Japan and Massachusetts Institute of Technology, Department of Materials Science and Engineering, Cambridge, Massachusetts 02139, United States

^now at: Faculty of Physics and Astronomy, Advanced Transmission Electron Microscopy, Ruhr University Bochum, 44801 Bochum, Germany; and Research Center Future Energy Systems, Ruhr University Bochum, 44801 Bochum, Germany

Table of Figures

Figure S1. XRD spectrum and AFM images of as-grown 50 nm thick STNi films.

Figure S2. EDS analysis of plan-view liftouts of the 150 nm thick STNi and STNNi films

Figure S3. EDS analysis of cross-section liftouts of 150 nm thick STNi and STNNi films

Figure S4. cAFM measurement of the Nb-doped SrTiO3 substrate.

Figure S5. Determination of the cluster size of the as-grown STNi film

Figure S6. LDOS mapped by STS illustration in sample bias case.

Figure S7. DFT calculations for cases of Ni incorporation into the STO unit cell.

Figure S8. STEM analysis of NiO_X cluster inclusions in both STNi and STNNi films

Figure S9. STM and STS of the STNi film after 400 °C annealing for 20 minutes.

Figure S10. Exsolution STM image of 300 nm by 300 nm.

Figure S11. Tip calibration for the STO measurement on Au(111) surface.

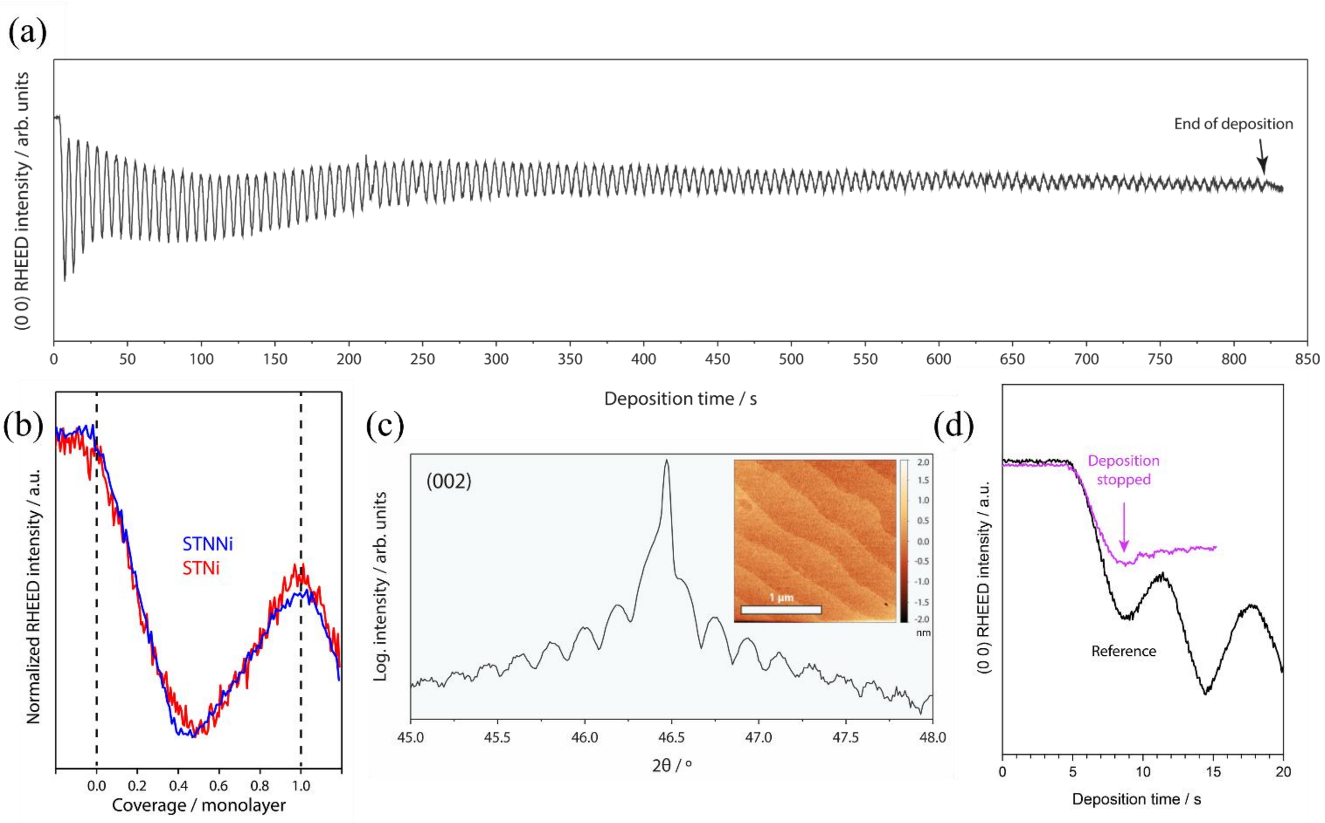

Figure S1. As-grown 50 nm thick STNi and STNNi films characterization and 0.5 monolayer STNi RHEED data. (a) STNi layer-by-layer type signatures recorded from the (0 0) specular spot of the RHEED pattern, (b) Close-up view of the first RHEED minimum during STNi and STNNi growth, (c) XRD in the (002) region showing strong overlap of the STNi thin film and substrate diffraction signal with thickness fringes demonstrating well-defined interfaces. Inset: representative AFM imaging of the STNi surface morphology showing a smooth surface morphology. (d) RHEED data for the deposition of 0.5 monolayer of STNi in comparison to data obtained during the deposition of a thicker film.

X-ray diffraction (XRD) analysis reveals that the thin film peak is partially superimposed with the substrate peak, while defined Laue-oscillation are in the vicinity of the thin film peak are apparent. The diffraction pattern indicates the growth of a coherent thin film with well-defined interfaces, whereas no indications for secondary phase formation become visible in the investigated 2θ range.

RHEED-monitored PLD deposition of 0.5 monolayer STNi is shown in Figure S1(d). The deposition is terminated at the first intensity minimum, corresponding to the intended 50% surface coverage. For comparison, RHEED data acquired during the growth of a thicker film are also included. An offset has been applied to facilitate comparison.

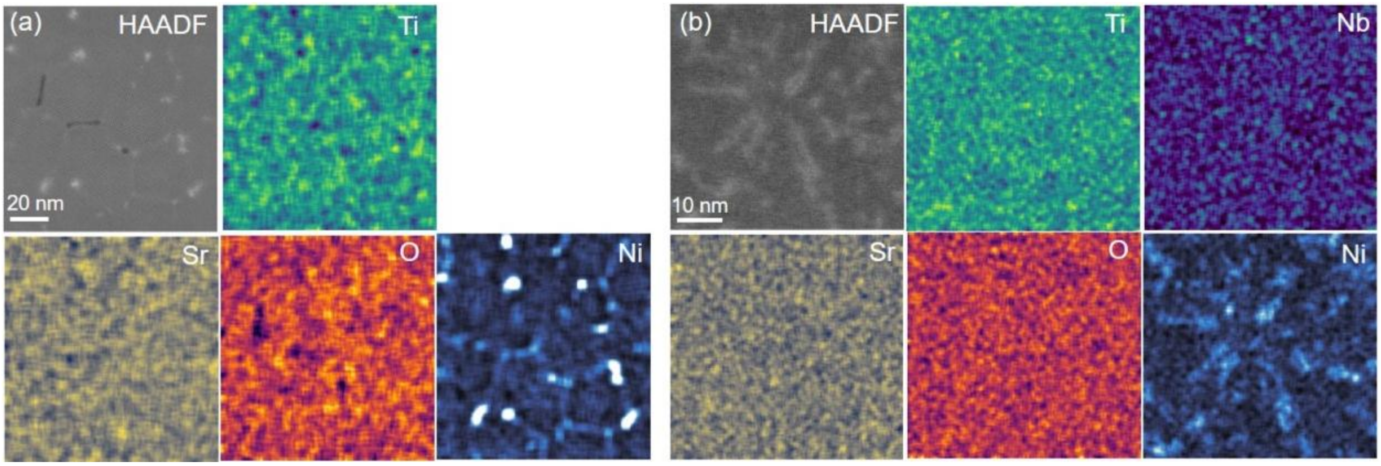

Figure S2. EDS analysis of plan-view liftouts of the 150 nm thick (a) STNi and (b) STNNi thin films. Note that the bright contrast in the STEM images corresponds to regions of increased Ni content. (a) is reproduced from Jennings et al.[1], licensed under CC BY 4.0.

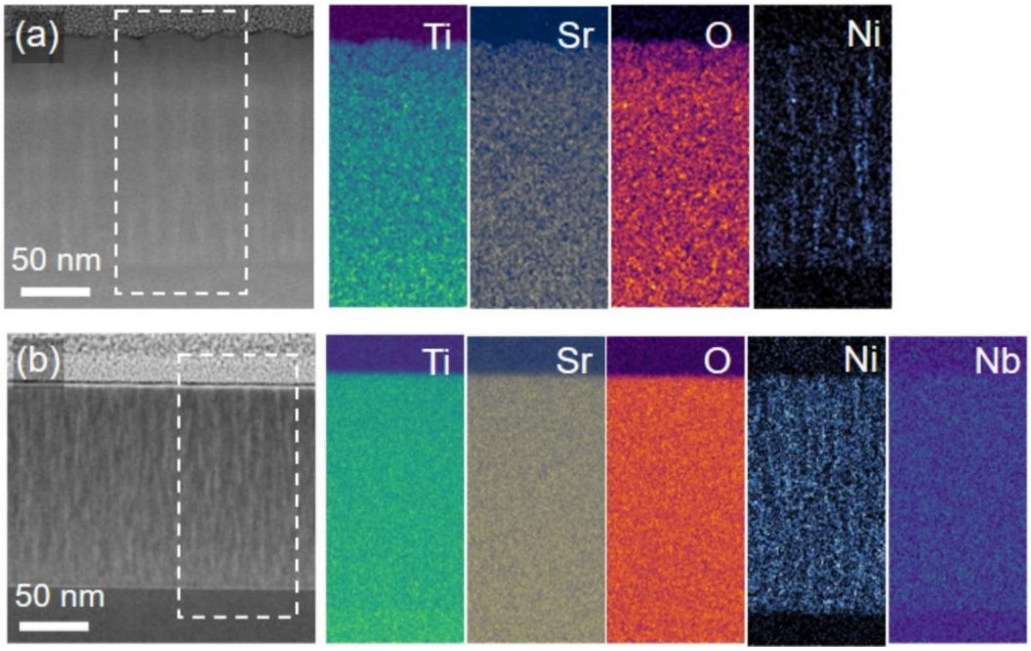

Figure S3. EDS analysis of cross-section liftouts of 150 nm thick STNi (a) and STNNi (b) thin films. The Ni-rich columns are observed to reach through the full thickness of the film for both samples.

The Nb-doped STO substrate is first characterized using cAFM to understand the surface quality and conductivity for the later STM measurements. Their morphology, scan profile across the atomic steps, and the conductivity behavior are shown in Figure S4.

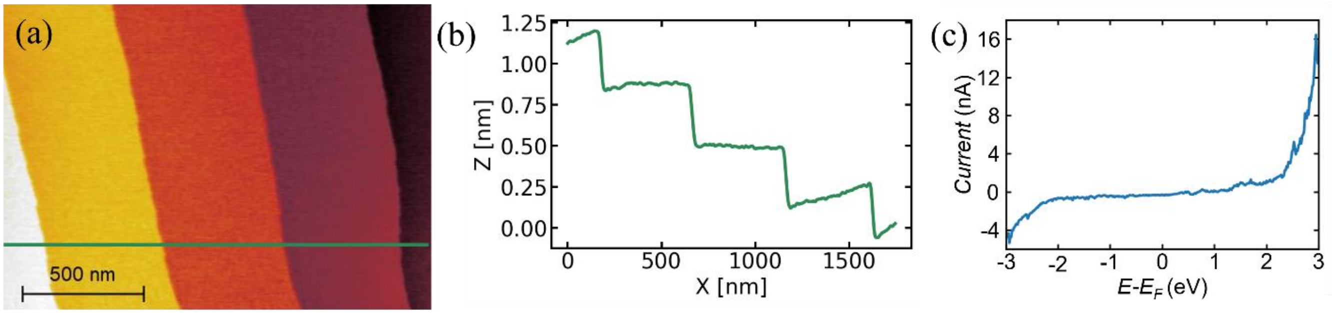

Figure S4. Contact-mode cAFM measurement of the Nb-doped $SrTiO_3$ substrate. (a) Topography showing five atomic steps (b) scan profile extracted from the green line from (a), using second-order parabolic background subtraction (c) I-V performance as a conductivity test using the cAFM with PPP-CONTPt cantilever at 2nN.

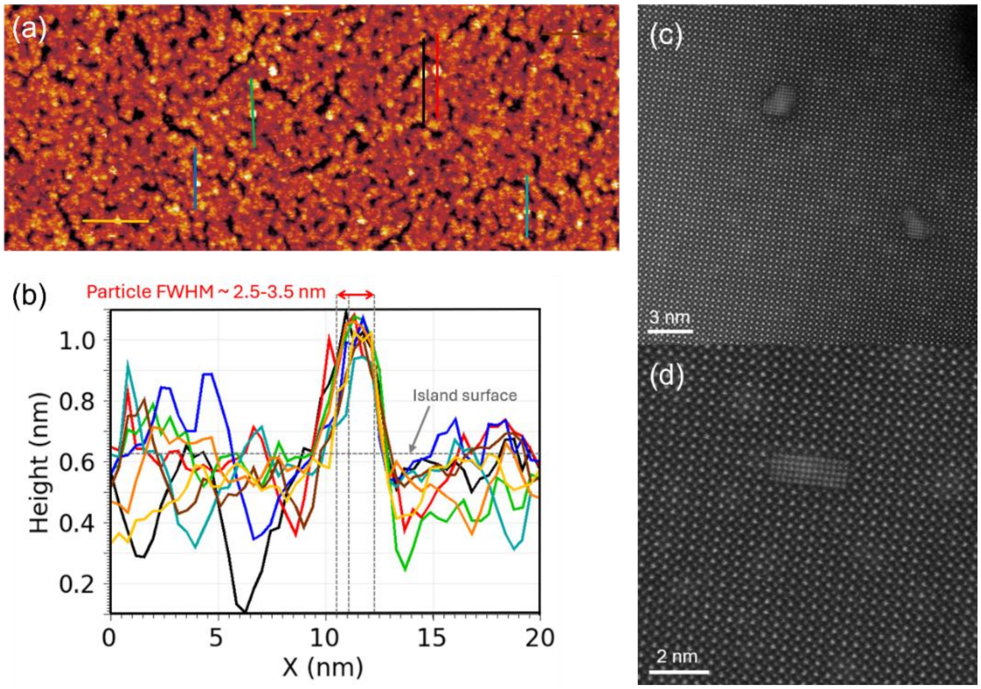

Figure S5. Determination of the cluster size of the as-grown STNi film. (a) STM topography of the region as in Figure 3(a). (b) The scan profiles of the particles extracted from (a), concluding cluster FWHM diameters in the range of 2.5-3.5 nm. (c-d) STEM HAADF images showing the cluster size between 1.5-3 nm.

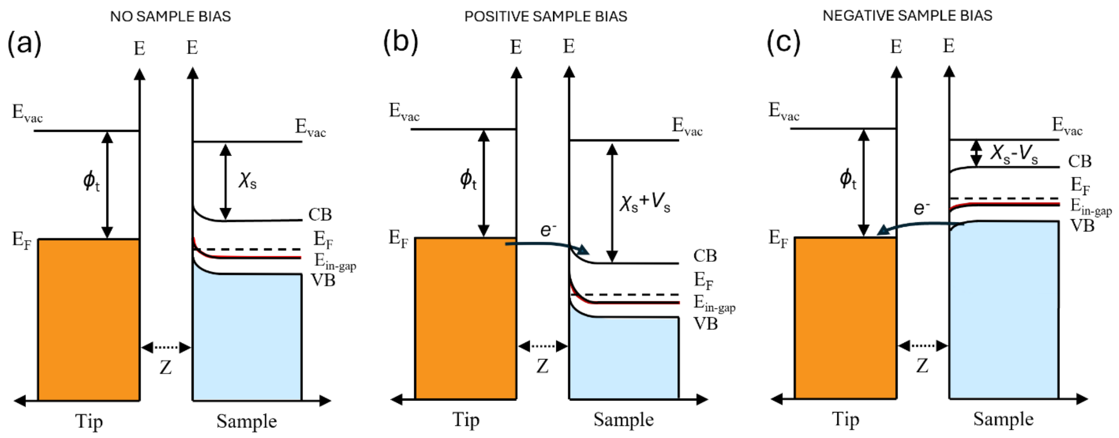

Figure S6. LDOS mapped by STS illustration in sample bias and a W tip case. (a) no sample bias (b) positive sample bias (c) negative sample bias.

Density functional theory (DFT) calculations

The first-principles (ab initio) computations were performed within the linear combination of atomic orbitals (LCAO) approximation of the density functional theory (DFT) as implemented in CRYSTAL23[2,3]. The Gaussian basis sets with Hay and Wadt small core pseudopotential were applied for the description of Sr and Ti atoms[4], while the all-electron basis sets were used for oxygen[4] and Ni atom[5]. All basis sets are available online on the CRYSTAL Basis Sets Library web site[6]. The B1WC global hybrid DFT-HF exchange-correlation functional with standard fraction (16%) of Hartree-Fock (HF) exchange[3] was employed in these calculations. This functional was already proved to be reliable in the simulations of systems based on STO and is well suited for the calculations of the band gap[7–10].The supercell (SC) approach with the periodic boundary conditions was used to simulate defects in STO crystal. Defects were created in the SC 2x2x2 or 3x3x3 constructed from a cubic STO (space group SG 221) with the atoms in the following Wyckoff positions: Sr:1b(1/2,1/2,1/2), Ti:1a(0,0,0), and O:3d(1/2,0,0). SC 2x2x2 contains 40 atoms (8 Sr and Ti atoms each and 24 oxygen atoms), while SC 3x3x3 contains 135 atoms (27 Sr, 27 Ti, and 81 oxygen atoms) before defect creation. All calculations were performed for diamagnetic systems (all chemical bonds are closed) and in the framework of space group 1 (without taking symmetry into account).

Here, we performed the expected density of states as inferred from first principles calculations based on the linear combination of atomic orbitals (LCAO) approximation in Figure S7. When using SC 2x2x2, the concentration of Ni atoms is 12.5% and the oxygen vacancies 4.17% relative to the stoichiometric number of Ti and oxygen atoms, respectively. The calculations for different Ni incorporate cases, including (i) a substitutional Ni^{2+} ion replacing Ti^{4+} with nearby neutral oxygen vacancy in the strontium titanate, also called first nearest neighbor (1NN) sites (SC 2x2x2), (ii) Ni^{2+} ion replacing Ti^{4+} with furthest neutral oxygen

vacancy (SC 2x2x2), and (iii) Ni^{4+} ion with no oxygen vacancy replacing Ti^{4+} (SC 3x3x3, the Ni concentration is 3.7%). In the first two cases, Ni^{2+} replacing Ti^{4+}, creating oxygen vacancies, Vö, gives a result of complex defect cluster Ni''-Vö. The fully relaxed geometries of these two defect configurations, shown in Figure S7(a) and S7(b), indicate the in-gap states induced by complex defect clusters Ni''-Vö in different situations. Note that for SC 2x2x2 the energy of the system with the maximum distance (5.8 Å) between the Ni ion and the oxygen vacancy is 2 eV higher than for the system with the minimum distance (1.8 Å) between Ni and the oxygen vacancy. The in-gap states appear below the Fermi level, which is opposite to the *dI/dV* observed in Figure 4(b). The calculations reveal that indeed the presence of a Ni''-Vö defect cluster can create extra energy states (bands) inside the band gap, above the valence band top. These calculation results may narrate the link between the observed in-gap states and the defect clusters.

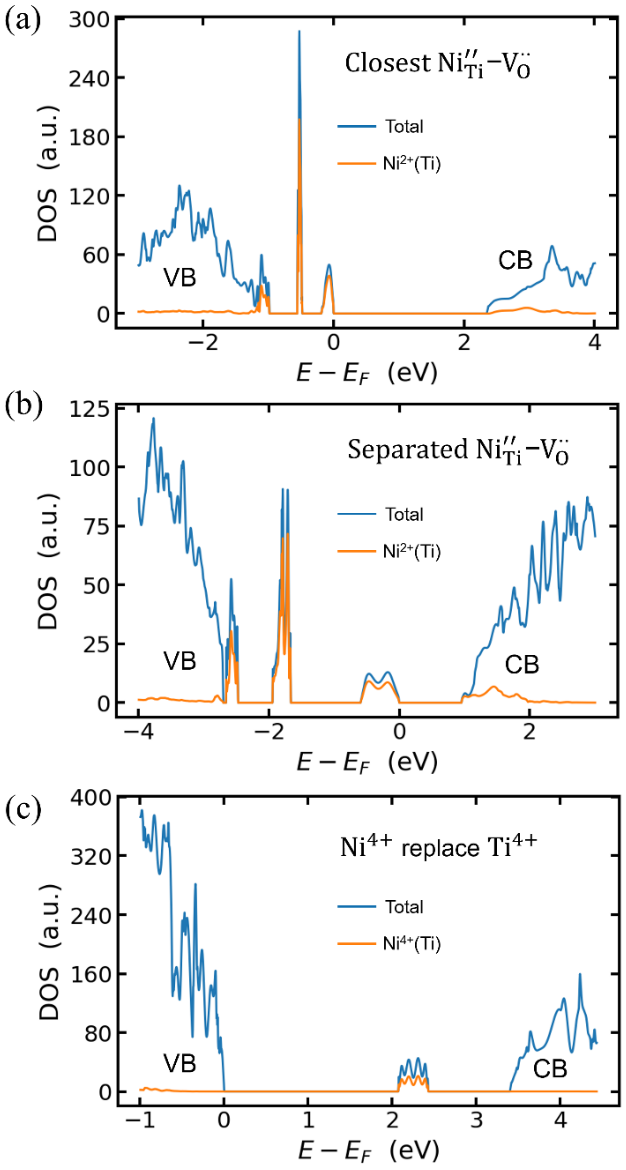

Figure S7. DFT calculation for the projected ($Ni^{2+/4+}$) and total electronic DOSs of STO. (a) a substitutional Ni^{2+} ion replacing Ti^{4+} with nearby neutral oxygen vacancy in the strontium titanate, also called first nearest neighbor (1NN) sites (SC 2x2x2). (b) Ni^{2+} ion replacing Ti^{4+} with the separated neutral oxygen vacancies in the SC 2x2x2. (c) Ni^{4+} ion with no oxygen vacancy replacing Ti^{4+} (SC 3x3x3).

Figure S8 shows Fast Fourier transformation (FFT) of the structure of NiO_X inclusions in the two films. In the STNi film, the FFT clearly shows a second set of reflections which correspond to the {200} planes in the NiO_x rocksalt lattice (with a measured lattice spacing of ~4.2 Å assuming an $SrTiO_3$ lattice spacing of 3.9 Å). The FFT of the STNNi film does not show clear reflections, likely due to the very small size of the inclusions in this case. By Fourier filtering using the expected positions of the NiO_x reflections, it can be seen that stronger intensity appears in regions of the Ni rich inclusions. This indicates that the lattice is expanded in these regions, and is consistent with the presence of small (~2 nm diameter) NiO_x inclusions. However, the exact phase of the inclusions cannot be definitively determined in this case.

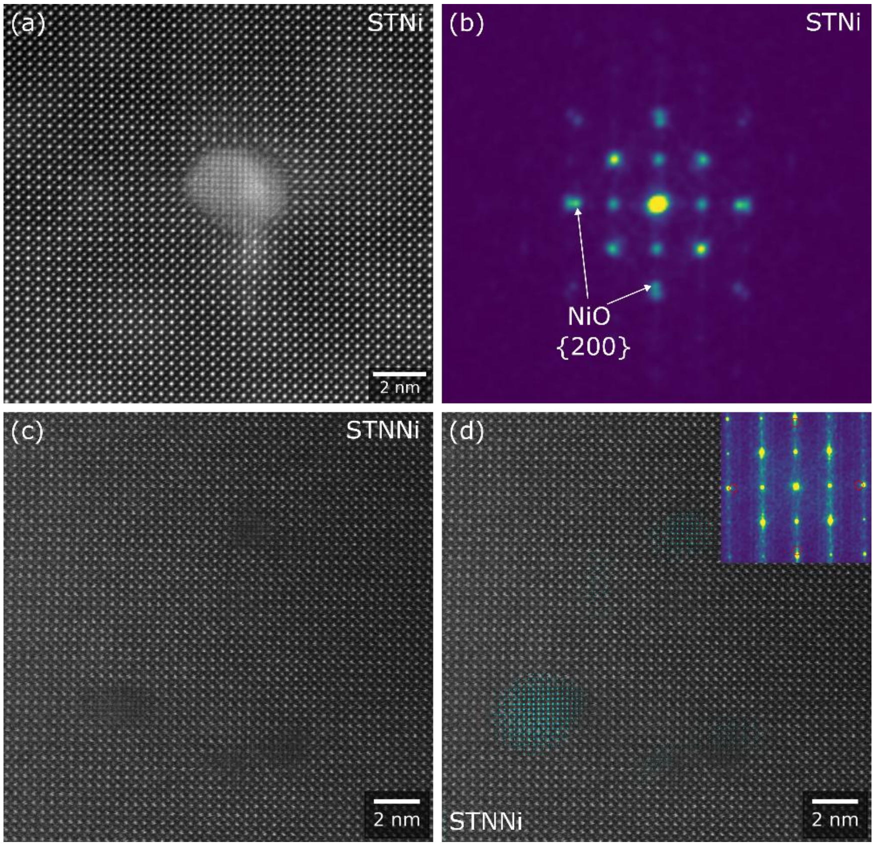

Figure S8. STEM analysis of NiO_X cluster inclusions in both STNi and STNNi films (a) HRSTEM HAADF image of an inclusion in the STNi thin film, along with (b) a fast Fourier transform of the region with the inclusion in (a). The main peaks in (b) can be attributed to the strontium titanate matrix, while a second set of reflections are present which correspond to the {200} planes of rocksalt NiO_x. (c) A corresponding HRSTEM HAADF image of inclusions in the STNNi film, along with (d) an overlayed fourier filtered image utilizing the {200} rocksalt NiO_x reflections (indicated by red circles in inset FFT). While the reflections are not clear in the FFT in (d), the Fourier filtered image shows stronger signal in the regions of inclusions, indicating these are regions where the lattice is expanded compared to the strontium titanate matrix. This expansion is consistent with the presence of small (~2nm diameter) NiO_X inclusions.

After 400 °C annealing for 20 minutes, the surface shows a nearly identical surface as pre-annealed condition, as shown in Figure S9. The *dI/dV* spectrum gives a consistent, sharp in-gap state on the particles as in the as-grown STNi, indicating that the Ni is still incorporated with the $SrTiO_3$ perovskite lattice.

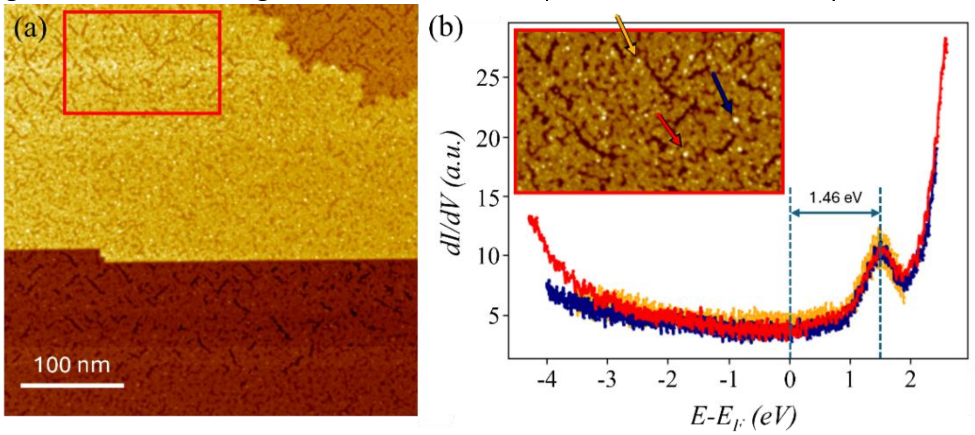

Figure S9. STNi film after 400 °C annealing for 20 minutes. (a) STM image of 300 nm by 300 nm. (b) STS and dI/dV spectra on the particles (c) STS and dI/dV spectra on the film.

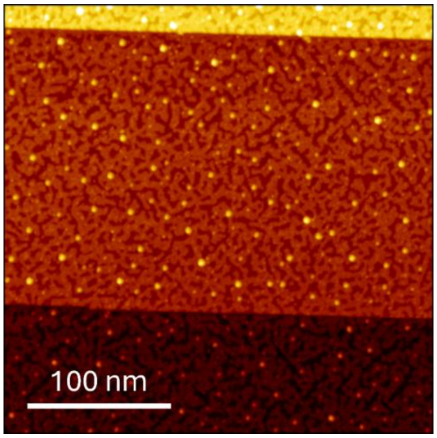

Figure S10. Exsolution STM image of 300 nm by 300 nm showing exsolved particles on three individual atomic stages without a regular pattern of the film.

Exsolved particle diameter estimation using the quantum dot equation

The exsolved Ni particles could be considered as quantum dots while performing STS. With the measured $E_{n=1}$=0.35 eV, the radius of the quantum dot can be calculated to be:

$$E_n = \frac{n^2\pi^2\hbar^2}{2m_{eff}R^2}$$

, where *h* is the Planck constant, m_{eff} is the effective mass, and *R* is the radius of the quantum dot. Here, we use the first oscillation peak 0.35 eV as $E_{n=1}$, m_{eff} for Ni is 0.19 m_e.[11]

$$R = \sqrt{\frac{1^2\pi^2\hbar^2}{2m_{eff}*0.35*1.6*10^{-19}}} = \sqrt{\frac{(3.14^2)(6.626\times10^{-34}/2\times3.14)^2}{2*(0.19\times9.11\times10^{-31})*0.35*1.6*10^{-19}}} \sim 2.4 \text{ nm}$$

Therefore, the estimated diameter of the exsolved Ni nanoparticle is about 4.8 nm.

Calculation of the Ni amount in the B-site of the perovskite lattice

All calculations are based on the number of Ni atoms within a 200 x 200 nm^2 area. The theoretical number of Ni is calculated with 5% of Ni doping on the B-site in a 75% coverage sub-monolayer (coverage acquired from STM images in section 2.2). The experimental calculation assumes the nanoclusters (as-grown) and nanoparticles (after exsolution) are in the shape of semi-ellipsoids, with volume given by the area (πr^2) multiplied by $\frac{2}{3}$ and the experimental particle height h (extracted from the STM scan profiles). The surface coverage in a given range equals the total area of all clusters/particles (sum of πr^2) within that range. Therefore, in the calculations 2) and 3), the particle volume is estimated as the product of area (200 x 200 nm^2 x coverage) and $\frac{2}{3}h$. By correlating this volume with the material density, the number of Ni atoms can be estimated.

1. Theoretical Ni number:
   (75% coverage in the as-grown sub-monolayer) x (5% Ni concentration on B-site per cm^3) x (height of the film 4Å) x $(200\ nm)^2$

   (0.75) x (8.4×10^{20} cm^{-3}) x (4×10^{-8} cm) x $(2\times10^{-5}\ cm)^2$ = **10080** (Ni atoms)

2. Exsolved Ni number:
   Area: (1.1% coverage of exsolved particles) x $(200\ nm)^2$
   Volume: (2/3 semisphere shape correction) x Area x (averaged height ~4Å from STM scan profile)
   Concentration of Ni metal: (6.022×10^{23} atoms/mol) x (8.908 g/cm^3) / (58.69 g/mol) = 9.14 x 10^{22} atoms/cm^3

   0.011 x $(2\times10^{-5}cm)^2$ x 2/3 x (4×10^{-8}cm) x (9.14×10^{22} atoms/cm^3) = **10724** (Ni atoms)

3. Ni in the as-grown cluster:
   Area: (0.35% coverage of culusters) x $(200\ nm)^2$
   Volume: (2/3 semisphere shape correction) x Area x (averaged height, Figure S5 (b))
   Ni Concentration of NiO: (6.022×10^{23} atoms/mol) x (6.67 g/cm^3) / (74.7 g/mol) = 5.377 x 10^{22} atoms/cm^3
   Notably, NiO is chosen as the upper limit reference because it has the highest Ni atom density compared to NiO_x and $\mathrm{Ni}_{\mathrm{Ti}}''$-$\mathrm{V}_{\mathrm{O}}^{\cdot\cdot}$ -enriched STO.

   0.0035 x $(2\times10^{-5}cm)^2$ x 2/3 x (4×10^{-8}cm) x (5.377×10^{22} atoms/cm^3) = **2007** (Ni atoms)

   The calculation indicates two statements (i) around 20% (2k/10k) of the Ni was formed as clusters in the as-grown deposition, while the rest of the Ni was exsolved from the perovskite lattice, nearly 80%.

To ensure the imaging quality, tip artifact needs to be carefully evaluated. In our group, we used on Au (111) crystal for tip conditioning and imaging as a standard procedure before moving to the sample for measurement. For oxide surfaces, the tip stability at a higher bias requires further tip conditioning. The STM/S presented in Figures 2-5 for STNi sample is measured with the same tip calibrated on Au 111 crystal and shown in Figure S11. The Au crystal is sputtered with Ar partial pressure of 2x10^{-5} mbar and annealed in UHV, with base pressure ~9x10^{-11} mbar.

The reference Au image, Figure S11(a), shows a step edge close to theoretical value of Au atomic layer of 2.3 Å, indicating a precise Z-axis calibration. The parallel ridge patterns on the same atomic terrace confirm the herringbone structure of the Au 111 surface reconstruction[12,13]. More relevant to this work is that the sub-nanometer clusters exhibiting at the kinks can be imaged nicely. These clusters represent the impurities, which segregate to the kink/elbow of herringbones during sputter annealing cycles due to energy preference[14], as can be seen in Figure S11(a,b). A zoom-in image of 10 nm by 10 nm is presented in Figure 3(c), where atomic resolution with its height profile shows individual atoms and their arrangement of the herringbones (brighter ridges).

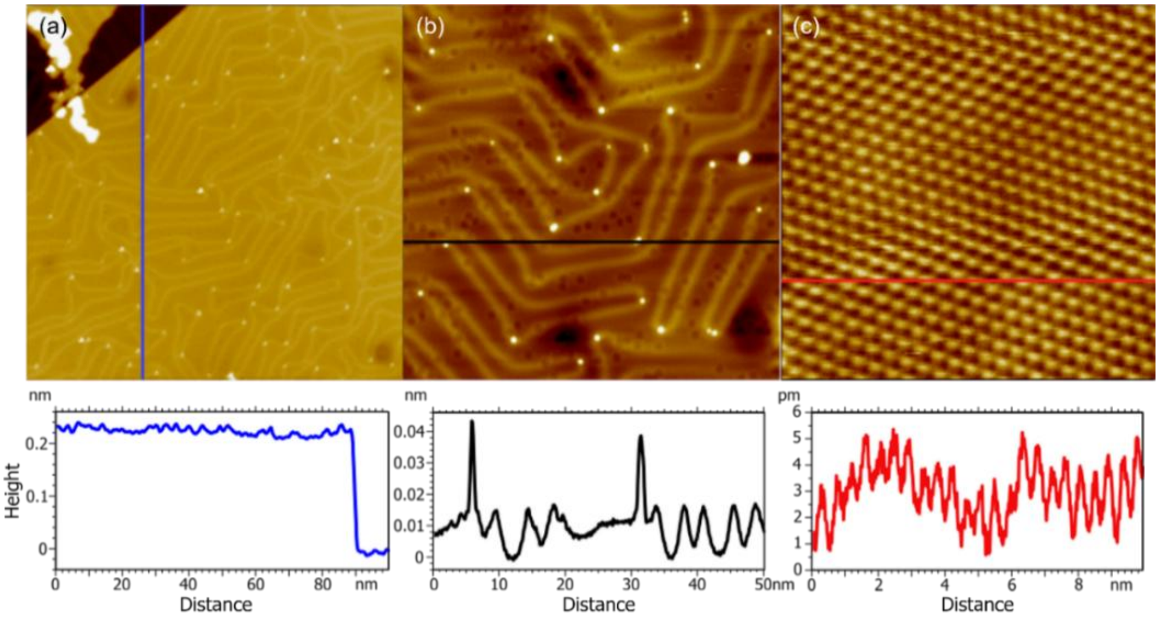

Figure S11. Tip calibration for the STO measurement on Au(111) surface. The same tip was subsequently used for all STM and STS measurements on the STNi sample, presented in Figures 2-5 and Figure S9-10.

References

1. D. Jennings, M. L. Weber, A. Meise, et al., "Direct Atomic-Scale Investigation of the Coarsening Mechanisms of Exsolved Catalytic Ni Nanoparticles," *Nature Communications 16*, no. 1 (2025): 6830, https://doi.org/10.1038/s41467-025-61971-z.
2. A. Erba, J. K. Desmarais, S. Casassa, et al., "CRYSTAL23: A Program for Computational Solid State Physics and Chemistry," *Journal of Chemical Theory and Computation 19*, no. 20 (2023): 6891–6932, https://doi.org/10.1021/acs.jctc.2c00958.
3. R. Dovesi, V. R. Saunders, C. Roetti, et al., "CRYSTAL23 User's Manual," *CRYSTAL23 User's Manual*, University of Torino: Torino, Italy 2022.
4. S. Piskunov, E. Heifets, R. I. Eglitis, and G. Borstel, "Bulk Properties and Electronic Structure of SrTiO3, BaTiO3, PbTiO3 Perovskites: An Ab Initio HF/DFT Study," *Computational Materials Science 29*, no. 2 (2004): 165–178, https://doi.org/10.1016/j.commatsci.2003.08.036.
5. M. F. Peintinger, D. V. Oliveira, and T. Bredow, "Consistent Gaussian Basis Sets of Triple-Zeta Valence with Polarization Quality for Solid-State Calculations," no. 34 (2013): 451–459.
6. CRYSTAL Basis Sets Library. Available online:, 2025.
7. L. L. Rusevich, G. Zvejnieks, and E. A. Kotomin, "Ab Initio Simulation of (Ba,Sr)TiO3 and (Ba,Ca)TiO3 Perovskite Solid Solutions," *Solid State Ionics 337* (2019): 76–81, https://doi.org/10.1016/j.ssi.2019.04.013.
8. G. Zvejnieks, L. L. Rusevich, D. Gryaznov, and E. A. Kotomin, "Interface-Induced Enhancement of Piezoelectricity in the $(SrTiO_3)_m/(BaTiO_3)_{M-m}$ Superlattice for Energy Harvesting Applications," *Physical Chemistry Chemical Physics 21*, no. 42 (2019): 23541–23551, https://doi.org/10.1039/C9CP04086B.
9. M. Tyunina, L. L. Rusevich, M. Savinov, E. A. Kotomin, and A. Dejneka, "Dielectric Behaviour of Nitrogen Doped Perovskite $SrTiO_{3-\delta}N_{\delta}$ Films," *Journal of Materials Chemistry C 11*, no. 47 (2023): 16689–16698, https://doi.org/10.1039/D3TC03757F.
10. L. L. Rusevich, E. A. Kotomin, G. Zvejnieks, et al., "Effects of Al Doping on Hydrogen Production Efficiency upon Photostimulated Water Splitting on $SrTiO_3$ Nanoparticles," *The Journal of Physical Chemistry C 126*, no. 50 (2022): 21223–21233, https://doi.org/10.1021/acs.jpcc.2c05993.
11. Y. Nishimura, M. Kakeya, M. Higashiguchi, et al., "Surface Electronic Structures of Ferromagnetic Ni(111) Studied by STM and Angle-Resolved Photoemission," *Physical Review B 79*, no. 24 (2009): 245402, https://doi.org/10.1103/PhysRevB.79.245402.
12. P. Li, and F. Ding, "Origin of the Herringbone Reconstruction of Au(111) Surface at the Atomic Scale," *Science Advances 8*, no. 40 (2022): eabq2900, https://doi.org/10.1126/sciadv.abq2900.
13. F. Hanke, and J. Björk, "Structure and Local Reactivity of the Au(111) Surface Reconstruction," *Physical Review B 87*, no. 23 (2013): 235422, https://doi.org/10.1103/PhysRevB.87.235422.
14. N. C. Bartelt, and K. Thürmer, "Structure and Energetics of the Elbows in the Au(111) Herringbone Reconstruction," *Physical Review B 104*, no. 16 (2021): 165425, https://doi.org/10.1103/PhysRevB.104.165425.
